# HU-EnviroGrids: A Gridded Environmental Dataset for Spatial Analysis and Modelling at country scale in Hungary

**DOI:** 10.1038/s41597-026-07604-6

**Published:** 2026-06-09

**Authors:** Katalin Takács, János Mészáros, László Pásztor, Annamária Laborczi, Zsófia Bakacsi, Brigitta Szabó, Tünde Takáts, Gábor Szatmári

**Affiliations:** 1 Institute for Soil Sciences, HUN-REN Centre for Agricultural Research, Budapest, Hungary; 2 Doctoral School of Earth Sciences, Faculty of Science, Eötvös Loránd University, Budapest, Hungary

## Abstract

There is a growing demand for comprehensive environmental information driven by increasingly complex environmental challenges. Addressing these issues requires multidisciplinary approaches supported by harmonized datasets derived from diverse data sources. We developed HU-EnviroGrids, a spatially exhaustive, gridded environmental dataset covering the entire territory of Hungary. The dataset was created using a multidisciplinary data integration approach that harmonized diverse data sources across multiple environmental domains, such as topography, hydrology, climate, biosphere & land surface and pedosphere. The derived quantitative and qualitative environmental variables were transformed into a common reference system, providing a consistent spatial coverage at a 100 × 100 m spatial resolution across Hungary. This dataset provides foundational inputs to a wide range of applications for applied research in ecology, hydrology, climatology, agriculture, forestry, and Earth system sciences. By making these data openly available, HU-EnviroGrids seeks to strengthen the scientific and practical basis for addressing complex environmental issues, and to foster more informed, data-driven decision-making at multiple scales.

## Background & Summary

There is a growing demand for comprehensive environmental information covering all major components of the Earth system, including the lithosphere, atmosphere, hydrosphere, biosphere, and anthroposphere. This demand is driven not only by the increasing scale and complexity of environmental challenges and crises, such as climate change, biodiversity loss, soil and land degradation, and food and water security, but also by the need for a deeper understanding of environmental processes and interactions^1^. Access to spatially exhaustive, reliable, and high-resolution environmental data is essential for advancing research, supporting decision-making, and addressing both current and emerging environmental issues^2,3^.

In parallel, the scientific landscape has evolved substantially over recent decades. Environmental research has become increasingly multi- and interdisciplinary, reflecting the complexity of the interactions between natural systems and human activities. No single discipline can fully address these multifaceted challenges. Instead, integrative approaches combining ecology, hydrology, climatology, agriculture, Earth system science, economics and the social sciences are required^4^. Such approaches rely on harmonized and comprehensive datasets that enable holistic analysis, robust modelling, and the development of evidence-based solutions.

The potential applications of environmental datasets are remarkably wide not only on an international scale, but also in Hungarian practice. These datasets have already been used in a variety of contexts, including mapping potential natural vegetation^5,6^, quantifying ecosystem services^7–10^, modelling the spread of invasive species^11,12^, monitoring land use and land cover change^13,14^, modelling agricultural productivity^15–18^, producing digital maps of soil properties and functions^19–21^, developing pedotransfer functions^22–25^, assessing soil and land degradation^26–29^, assessing extreme soil moisture conditions^30–32^, and supporting hydrological and hydrogeological modelling^33–36^. These datasets are valuable not only for researchers, but also for practitioners in agriculture, engineering, economics, landscape planning, and rural development, as well as for policy makers. Additionally, the demand for these datasets occurs across multiple spatial scales, ranging from local field and catchment levels to national, continental, and global levels. Furthermore, environmental information is increasingly sought by the wider public, reflecting a growing societal awareness and engagement with environmental issues.

Numerous global, regional and national initiatives have been launched to create comprehensive gridded spatial datasets containing a diverse array of environmental data^37–39^ or focusing on selected environmental domains such as topography^40,41^, hydrology^42,43^, climate^44,45^, biosphere & land surface^46,47^ and pedosphere^20,48^ for modelling purposes. Data cube technology provides analysis-ready, harmonized time series of spatially aligned, multidimensional Earth Observation (EO) data, and may additionally offers analysis tools and platform^49^. EO data cubes facilitate the transformation of satellite observations into meaningful biophysical and environmental variables^46^. An increasing number of operational EO data cube implementations now exist at multiple scales, including global (OpenDataCube^50^), continental (EcoDataCube^51^, Digital Earth Africa^52^) and national systems (Digital Earth Australia^53^, Swiss Data Cube^54^).

At the same time, new analytical and computational approaches including geospatial artificial intelligence (GeoAI) and spatial foundation models, are undergoing rapid development^55,56^. These methods depend on the availability of large, well-structured, and high-resolution datasets to reach their full potential^57^. Interdisciplinary and transdisciplinary research, which now plays a central role in understanding and responding to complex environmental challenges, similarly relies on such data to generate robust knowledge and actionable insights^58^.

HU-EnviroGrids^59^ is a national initiative that provides a spatially exhaustive, gridded environmental dataset covering the entire territory of Hungary at 100 × 100 m spatial resolution and explicitly optimized for the geographical and ecological characteristics of the Pannonian Basin and thus Hungary. By adopting the national coordinate reference system, HU-EnviroGrids ensures seamless interoperability with other domestic datasets and field observations of local users. The accuracy of the HU-EnviroGrids is underpinned by national ground truth datasets as several variables (related to temperature, precipitation, ground water level or soil) are derived from local observation networks and interpolated using specific methods incorporating expert knowledge, thereby offering higher local fidelity than continental or global products. Furthermore, HU-EnviroGrids is distinguished by its thematic breadth. While global initiatives often focus on single domain such as Geomorpho90m^60^ – topography, SoilGrids^48^ – soil, and CHELSA-BIOCLIM+^45^ – climate, this dataset integrates 147 quantitative and qualitative variables across five distinct domains – topography, hydrology, climate, biosphere & land surface, and pedosphere – including specialized indices, which are uniquely relevant for Hungary. The dataset consists of pre-harmonized, version-controlled raster stacks providing analysis-ready-data to multivariate modelling and machine learning workflows ensuring consistent spatial alignment across all environmental predictors. HU-EnviroGrids is intended to support a wide range of applications, from fundamental and applied research in ecology, hydrology, climatology, agriculture, forestry, and Earth system sciences, to practical decision-making and policy formulation.

Beyond its scientific and practical relevance, HU-EnviroGrids also aims to contribute to public awareness and understanding of environmental processes through open and easily accessible data. In addition, the dataset can support broader continental and global initiatives, such as the European Union’s Common Agricultural Policy, the United Nations Sustainable Development Goals, and the Convention to Combat Desertification. By making these data openly available, HU-EnviroGrids seeks to strengthen the scientific and practical foundations for addressing complex environmental issues, and to foster more informed, data-driven decision-making at multiple scales.

## Methods

Environmental processes and landscape dynamics result from interactions within the Earth system. Environmental modelling relies on the integration of diverse environmental covariates that represent the underlying physical processes driving biophysical and ecological phenomena. We compiled a set of environmental variables for HU-EnviroGrids that characterise the physical, climatic, biological, and pedological features of the Earth’s surface, thereby supporting a broad range of environmental modelling applications. These variables provide essential information for understanding and predicting processes that shape landscapes, influence ecosystems and mediate interactions among atmosphere, hydrosphere, biosphere and pedosphere. The following sections describe the main environmental domains and associated variables included in HU-EnviroGrids, their data sources, methods of generation, and their relevance as covariates in environmental modelling.

### Variables related to topography

Topography plays a crucial role in environment driving hydrological, geomorphological or ecological processes^41^. The form, orientation or roughness of the surface, the vertical position and specific land features have been shown to strongly affect local climate characteristics, drainage potential, erosion-deposition processes, soil formation, vegetation types or species distribution. We have selected a couple of terrain variables representing the vertical position, shape, orientation, and complexity of the terrain, as well as indices characterizing specific landform types and positions that are found to be useful in the different aspects of environmental modelling.

As quantitative information on morphometric variations of land surface can be provided by digital elevation models^61^ (DEM), we selected FABDEM v1.2^62^ (Forest And Buildings removed Copernicus DEM; https://doi.org/10.5523/bris.s5hqmjcdj8yo2ibzi9b4ew3sn) for the basis of the topographic variables in HU-EnviroGrids. The FABDEM v1.2 elevation dataset is based on COPDEM30^63^, which is a 30-metre spatial resolution digital surface model acquired by the TanDEM-X satellite, and recently found to be the best representation of the global terrain^64^. As COPDEM30 is a surface model, it measures the top of the natural features and anthropogenic surface objects. To produce a ‘bare-earth’ terrain model, the bias caused by the forests and the buildings were removed by machine learning techniques^65^. The terrain derivatives provided are considered primary topographic attributes, which were calculated directly from FABDEM v1.2 using the relevant tools of SAGA GIS^66^ *Terrain Analysis* and *Spatial and Geostatistics* modules.

#### Vertical position

Altitude can be extracted directly from elevation data. As the vertical reference datum of COPDEM30 and thus FABDEM v1.2 is EGM2008^67^, the absolute elevations are referred to this geoid. Relative elevations reflect the actual position to relatively its surrounding surface elevations providing a more comprehensive geomorphological context. We used the Standardized Height^68^ (SH), which is a scaled measure of relative altitude, the Valley Depth^66^ (VD), which characterizes the vertical difference between local ridgeline and actual altitude and the Mid-slope Position Index^68^ (MSPI), which represents the normalized distance from mid-slope to both ridge and valley direction for the HU-EnviroGrids.

#### Terrain gradient and shape

Several terrain shape-related variables can be derived from DEM. Slope is calculated as the rate of change in altitude in the direction of the steepest slope representing terrain gradient and given in HU-EnviroGrids in degrees^69^. Curvatures are defined as the second derivative of surface elevation and derived from slope and aspect properties. Profile Curvature (PC) measures the rate of change in altitude in the direction of the steepest slope or the flow line. General Curvature (GC) provides a standardized measure of surface curvature regardless of slope direction and indicates the overall convexity or concavity of the terrain^70^. Total Curvature (TC) means the magnitude of curvature of the surface itself, not in a particular direction of a line^71^. TC is always a positive value, unlike GC, which can be either positive (concave) or negative (convex) and emphasize rapidly changing, complex terrains with intense curvatures. Real Surface Area^69^ (RSA), which represent the actual 3D surface area, is also related to terrain gradient; as the terrain gets steeper the RSA increases compared to the 2D map projection area.

#### Terrain orientation

Aspect^72^ represents the orientation of the steepest slope; it is given in radian or it can be classified by cardinal directions: North: 0–22.5° and 337.5–360°; Northeast: 22.5–67.5°; East: 67.5–112.5°; Southeast: 112.5–157.5°; South: 157.5–202.5°; Southwest: 202.5–247.5°; West: 247.5–292.5°; Northwest: 292.5–337.5°. Diurnal anisotropic heat^73^ (DAH) is a simple approach to quantify the topographic effects on solar heating during daytime based on slope, aspect and sun position.

#### Terrain complexity

The variability of the altitudes within a specified area characterizes the spatial heterogeneity of the surface. The variability can be represented by statistical measures, here we used a neighbourhood area with a radius of 500 m to calculate the terrain complexity variables for HU-EnviroGrids. We derived the range of altitude values as Relief Intensity^74^ (RI), the variance as Surface Roughness^75^ (SR) and the root mean square as Terrain Ruggedness Index^76^ (TRI). Terrain complexity quantifies the surface topography and provides deeper insight into the spatial structure of the terrain.

#### Landform types

Several topographic variables are suitable to identify and characterize specific landforms based on elevation data. The Topographic Position Index^77^ (TPI) is the difference between the actual altitude and the average of the surrounding area indicating the local geomorphic features; here we used a neighbourhood area with a radius of 500 m. In contrast the Multiscale Topographic Position Index^77^ (MSTPI) integrates the TPI values calculated across multiple spatial scales and can capture the hierarchy of landform features in the landscape. Multi-resolution Valley Bottom Flatness (MRVBF) quantifies valley bottoms across multiple scales based on lowness (altitude) and flatness (slope variance). Complementary to MRVBF, the Multi-resolution Ridge-Top Flatness (MRRTF) indicates ridge tops across multiple scales based on highness (altitude) and flatness (slope variance) likewise MRVBF^78^.

### Variables related to hydrology

Land surface parameters that are particularly relevant to hydrology provide insights into the spatial distribution of surface and subsurface water dynamics^79^. These variables can be used as proxies for modelling the spatial pattern of moisture conditions, erosion/deposition processes or groundwater interactions^80^. Surface water dynamics can be represented by secondary or compound topographic variables, which requires information not only on elevation, but also on drainage network and specific catchment area and provides opportunity for a process-based terrain characterization^81^.

For the basis of the hydrological variables related to surface hydrology in HU-EnviroGrids, we used FABDEM v1.2 to characterize topography and actual surface water bodies^82^ to correct the inconsistency between the drainage patterns derived from the DEM and the real runoff conditions. While there is no single algorithm that can deal optimally with depressions in the applied DEM^83^, we combined the commonly used methods^84–86^ to handle sinks and create a hydrologically correct DEM. Depressions were initially filled, then the stream network was burned into the DEM based on the rasterized version of the actual surface water bodies (hereafter referred as channel network). A one-pixel wide buffer was also burned around the channel network, but with smaller offset, to moderate the vertical drop caused by stream burning. To preserve continuous flow after the burning process, any new depressions that may developed were breached. DEM conditioning was carried out by the *Preprocessing* tools of the *Terrain Analysis* module in SAGA GIS. Hydrological variables were derived from the hydrologically corrected DEM using the relevant tools of *Channels* and *Hydrology* in the *Terrain Analysis* module of SAGA GIS.

#### Channel network and proximity metrics

Channel Network Base Level (CNBL) is derived by interpolating the altitude of the channel network. The Vertical Distance to Channel Network (VDCN) can be calculated as the vertical difference between the actual altitude and CNBL. The Horizontal Distance to Channel Network (HDCN) represents the Euclidean distance to the channel network^72^.

#### Wetness indices

The topographic control on soil moisture can be characterized by wetness indices. The Topographic Wetness Index^87^ (TWI) estimates water accumulation potential based on local catchment area and slope. The SAGA Wetness Index^88^ (SAGAWI) is a common variant of the TWI, but it is calculated using an iteratively modified catchment area in order to reduce the effects of very small slopes in relatively flat areas, where the TWI method tends to overestimate the wetness.

#### Erosion and mass movement metrics

The Stream Power Index^89^ (SPI) is a measure of the erosive power of surface runoff, and calculated based on slope and specific catchment area. The LS-factor is one of the components of the Universal Soil Loss Equation^90^ (USLE) representing the effect of topography on soil erosion based on slope length (L) and steepness (S). Several GIS-based adaptations were proposed for formulizing the LS-factor^91^, here we used the approach of Moore *et al*.^89^, that replaces slope length with specific catchment area and calculates slope steepness using the sinus function of the slope angle normalized by 9% reference slope. A maximum flow path threshold (100 m) was utilized to avoid enormous values of LS-factor at stream locations^92^. The Mass Balance Index^93^ (MBI) is derived from slope, curvature and VDCN representing areas with net erosion/deposition or balanced material transport.

#### Subsurface hydrology

Subsurface hydrological conditions are important not only for representing a major freshwater resource, but also due to their considerable impact on land surface processes and terrestrial ecosystems that makes groundwater table level a critical parameter in environmental modelling^94,95^. For HU-EnviroGrids we provide a groundwater table depth (GWTD) map representing mean groundwater levels in the growing season, which were originally served as the basis for developing nationwide irrigation strategy^96^. The shallow groundwater depth observations were retrieved from the Central Hydrological Database of the General Directorate of Water Management^97^ and were averaged in each year from April to October and then were calculated a long-term mean for the 1981–2013 period. The groundwater observation data were interpolated to a 100-metre grid by regression kriging^98^ using several environmental covariates (altitude, HDCN, long-term mean precipitation and evapotransporation, parent material, NDVI and physical soil characteristics).

### Variables related to climate

Climatic variables play essential role in environmental modelling as they affect a wide range of physical, biological and chemical processes and provide key inputs to ecological, hydrological, and agricultural models^45,99^. Long-term annual and seasonal means of climatic variables present an overview about the spatial patterns, the interannual variability and the seasonal contrast of environmental conditions and can serve as a reference for identifying climate anomalies of the target area^100^.

#### Temperature and precipitation

The data on temperature and precipitation is originated from the Meteorological Database of HungaroMet^101^ (https://odp.met.hu/), which provides homogenized climate data series for Hungary derived from ground observations of the HungaroMet stations back to 1971. Initially, the daily station data are quality controlled, homogenized and gap filled by the MASH method^102^, then interpolated to a regular grid with a spatial resolution of 0.1° using the MISH algorithm^103^.

#### Global radiation and potential evapotranspiration

The Global radiation data and Potential Evapotranspiration (ET_pot_) were retrieved from E-OBS^104^ (https://doi.org/10.24381/cds.151d3ec6). E-OBS is a European, high-resolution ensemble climate dataset^105^, which relies on the station data collected by the ECA&D initiative^106,107^ covering the period back to 1950. The daily observations are subject to quality control, then blended and homogenized^108^, and subsequently spatially interpolated to a regular grid at 0.25° and 0.1° resolution. The global radiation dataset was created by combining *in-situ* and satellite-based (CERES; Cloud and the Earth’s Radiant Energy System) observations in order to reduce the inhomogeneity resulted from significant space and time variations of sunshine duration measurements^109^. ET_pot_ is a multi-element climate index derived from E-OBS daily datasets using the Penman-Monteith formulation^110^.

#### Annual and seasonal averages

We used the daily, gridded surface climate variables – temperature, precipitation, global radiation and ET_pot_ – at 0.1° resolution to calculate the long-term averages for 1991–2020, which is the most recent climate normal period defined by WMO^100^. Initially, the annual and seasonal means (temperature and global radiation) and sums (precipitation and ET_pot_) were calculated based on the daily values, then the annual values were averaged to the 1991–2020 period. The seasons were defined according to the conventional meteorological classification, whereby spring comprises as March, April, and May (MAM), summer as June, July, and August (JJA), autumn as September, October, and November (SON) and winter as December (of the previous year), January, and February (DJF).

### Variables related to biosphere & land surface

Land surface conditions and variables related to biosphere play a fundamental role in environmental modelling due to their critical role in regulating ecological processes and ecosystem functions and directly influencing energy, water and carbon fluxes between land and atmosphere^111^. EO provides large-scale, high-resolution (spatial, spectral and increasingly temporal) data on the biosphere and land surface, thus enabling the monitoring of land surface and vegetation dynamics, highlighting specific land surface and vegetation features and providing essential inputs to various land surface models^112,113^. Spectral indices can be derived from multi- or hyperspectral EO data focusing on the different domains of Earth system such as vegetation, water, soil or urban surfaces^114^.

#### LandSat spectral bands

Satellite imagery showing land surface in bare soil state can be derived from multi-temporal satellite images by aggregating the reflectance of selected exposed or vegetation-free surfaces^115^. For HU-EnviroGrids we used long-term and cloud-masked LandSat-4, −5, −7, −8 and −9 satellites’ Level-2 surface reflectance imagery (courtesy of the U.S. Geological Survey; Landsat 4–5: https://doi.org/10.5066/P9IAXOVV, Landsat 7: https://doi.org/10.5066/P9C7I13B, Landsat 8–9: https://doi.org/10.5066/P9OGBGM6) to generate bare soil composites. During the processing stage, the spectral bands were harmonized across different generations of Landsat satellites and sensors, therefore solely the Blue, Green, Red, Near Infrared (NIR), Shortwave Infrared (SWIR1 and SWIR2) bands were retained in the final composite images. A two-step masking process was employed to filter bare-soil pixels in all satellite images. Initially a mask layer was created to differentiate agricultural fields from any other land-use areas based on the Ecosystem Map of Hungary^116^. Secondly, an additional mask layer was computed, representing dry, bare soil pixels based on the Normalized Difference Vegetation Index (NDVI) and the Normalized Burn Ratio (NBR) spectral indices. The NBR was calculated as the normalized difference of NIR and SWIR2 bands. Threshold values were identified empirically for both indices. In the final workflow, pixels with NDVI values less than 0.25 and NBR values less than 0.2 were kept as bare soil surface. Following the implementation of masking, the final image was generated by mosaicking the two median images, one representing the filtered bare soil pixels, whilst a second for all other land-use surfaces. Google Earth Engine^117^ was employed to collect and pre-process Landsat satellite imagery and to derive the bare soil images. Pixels with reflectance values outside of the range of 0 to 1 were consistently masked through all of the spectral bands. The resulting data gaps and internal missing values were filled using a spline interpolation via *Close gaps* tool of the *Grid* module in SAGA GIS to provide complete and seamless coverage of the entire area of Hungary. A tension threshold of 0.01 was applied to the interpolation to ensure smooth transition while preserving the local characteristics of the observed reflectance.

#### Spectral indices

Several spectral indices, related to the plant-soil-water system were derived for HU-EnviroGrids based on the generated bare soil composites, for details see Table 1. Vegetation indices have been demonstrated to be a useful tool for assessing soil properties^118^ on the surface, as well as for indirect assessment through vegetation conditions. Consequently, three such indices were incorporated into HU-EnviroGrids: the NDVI^119^ is one of the most widely used vegetation indices quantifying vegetation greenness, health and productivity and monitoring of terrestrial ecosystems in space and time^114,120,121^, and it is calculated based on the normalized difference of the NIR and the Red bands. The Transformed Vegetation Index (TVI) and the Soil Adjusted Total Vegetation Index (SATVI) are further developed versions of NDVI. The TVI uses the normalized differences of the same spectral bands as NDVI, coupled with a square-root transformation and an additional constant value of 0.5. Slightly modified versions are also present in the literature^122,123^, we implemented the formula published in the latter form. SATVI uses SWIR band instead of NIR to increase the sensitivity to water content and enhance the observation of senescent vegetation^124^ and it is compiled with a soil adjustment factor (L; values go from 0 for dense vegetation to 1 for sparse vegetation) based on the concept of SAVI^125^ (Soil Adjusted Vegetation Index). TVI was found to have strong spatial and temporal correlation with green biomass and vegetation water content, especially during spring, at the time of early stages of vegetation growth^119^. On the other hand, SATVI was found to be sensitive to the detection of both green and dry (senescent) vegetation, especially in rangelands in semi-arid or arid ecosystems. Soil spectral characteristics, even only in the visible (blue-green-red) range, may be an important parameter in soil classification or quantification of colour-related parameters, e.g., soil organic content^123,126^. The Brightness Index (BI) was also incorporated into the dataset, and calculated as the function of Red and Green bands^127^. The Normalized Difference Salinity Index (NDSI) was included to address challenges caused by soil salinization that present also in Hungarian agricultural soil^128^. The NDSI was computed as the normalized difference of Red and NIR bands^129^. Both the wetness conditions of the land surface and the biosphere can be examined using water-related spectral indices. Here we used the Land Surface Water Index (LSWI), which has improved sensitivity to detect moisture condition of leaf, canopy and soil, and vegetation status, especially in croplands and wetland ecosystems^130^. LSWI was calculated based on the normalized difference of NIR and SWIR bands.

**Table 1:** Vegetation indices and formulas.

Index	Abbreviation	Formula
Normalized Difference Vegetation Index^119^	NDVI	${NDVI}=\frac{{NIR}-{RED}}{{NIR}+{RED}}$
Transformed Vegetation Index^119,122^	TVI	${TVI}=\left(\sqrt{\frac{{NIR}-{RED}}{{NIR}+{RED}}+0.5}\right)\times 100$
Soil Adjusted Total Vegetation Index^124^	SATVI	${SATVI}=\frac{{SWIR}1-{RED}}{{SWIR}1+{RED}+L}\times \left(1+L\right)-\frac{{SWIR}2}{2}$
Brightness Index^127^	BI	${BI}=\frac{\sqrt{({{RED}}^{2})+({{GREEN}}^{2})}}{2}$
Normalized Difference Salinity Index^129^	NDSI	${NDSI}=\,\frac{{RED}-{NIR}}{{RED}+{NIR}}\times 100$
Land Surface Water Index^130^	LSWI	${LSWI}=\frac{{NIR}-{SWIR}1}{{NIR}+{SWIR}1}$

#### Long-term and short-term remote sensing layers

We generated two types of remote sensing based data layer sets: one long-term layer set including the six harmonized satellite spectral bands in bare soil condition and the additional six layers of spectral indices for the period 1991–2020 corresponding to the climatic variables providing a baseline for consistent long-term environmental assessments. Furthermore, short-term layer sets with the same spectral bands and indices were also created for the entire 30-year period, which was divided into 5-year segments and extended to present to support dynamic modelling and monitoring changes in land management and degradation.

### Variables related to pedosphere

The pedosphere is defined as the soil mantle of the Earth, representing the intersection of the lithosphere, atmosphere, hydrosphere, and biosphere. Soils play a key role in Earth systems as acting like a dynamic interface between the different spheres of Earth. Accurate information on soil properties gives essential inputs for modelling water, energy and carbon balances, environmental risk assessment, crop modelling and yield prediction, food security, climate change adaptation and mitigation^131^. Soils provide a wide range of ecosystem services at different scales for the terrestrial ecosystems^48,132^.

#### Soil texture

Soil texture is defined as the relative proportion of different size of mineral particles in soils. While soil texture influences the interaction of land with water, climate, vegetation and biogeochemical processes, it is a fundamental physical soil property. For HU-EnviroGirds, the soil texture data is provided by the DOSoReMI.hu project^20^ (https://dosoremi.hu/) which is a collection of country-wide spatial soil information in the form of map products with a 100 m spatial resolution, supplied with the estimation of uncertainty. The soil texture maps of DOSoReMI.hu were created based on the soil observation data of particle size fractions (clay, silt and sand) of the Hungarian Soil Information and Monitoring System^133^ (SIMS) using regression kriging^134^. The prediction of particle size fractions was performed at standard depth intervals specified by GSM.net^135^ (0–5 cm, 5–15 cm, 15–30 cm, 30–60 cm, 60–100 cm, 100–200 cm). In HU-EnviroGirds, we provide soil texture class information for the 0–15 cm depth interval to capture the biologically active zone and the rooting depth of several plants, which is in the focus of most ecological and agricultural analysis. Soil texture classes for the 0–15 cm depth interval was derived based on the weighted average of clay, silt and sand fractions of the 0–5 cm and the 5–15 cm layers and then the results were classified into 12 distinct classes according to the USDA system^136^.

#### Soil type

Soil type is a composite indicator which is based on a classification system that takes into account the physical, chemical and biological characteristics and summarizes our thematic knowledge about soils^137^. Data on soil type classes were provided also by the DOSoReMI.hu project^20^ with a 100 m spatial resolution. The soil type map was created based on the harmonized observations of agricultural lands (SIMS; MARTHA^138^) and forests sites^139^. The prediction of soil type classes was carried out by the combination of multiresolution segmentation of environmental predictor variables and a two-level, multi-step sequential classification using CART (Classification and Regression Trees), RF (Random Forest) and ANN (Artificial Neural Network)^140^. The soil type map of HU-EnviroGrids comprises 41 soil type classes according to the Hungarian Soil Classification System, which is based on a genetic approach.

#### Parent material

From a pedological perspective parent material refers to the material from which a soil develops through weathering, translocation or accumulation processes^136^. The parent material represents the geological context of the landscape and the lithological factor of the soil forming processes and strongly influences directly many soil characteristics (mineral composition, chemical properties, texture and nutrient content) and indirectly the terrestrial ecosystems^141^. The parent material data for HU-EnviroGrids were provided by Bakacsi *et al*.^142^, in which parent material was defined according to the widely known and accepted FAO classification system^143^ and derived based on the lithology and the facies of the geological formations assessed by the Geological Map of Hungary^144^ (https://inspire-geoportal.ec.europa.eu/srv/api/records/7d29a580-7ea4-4bb1-8aa5-c7159a4f8c32).

### Common grid system and data harmonization

In order to compile a spatially consistent dataset, the various thematic environmental variables were transformed into a common grid system. For HU-EnviroGrids we used the Hungarian Unified National Projection System (HD72/EOV, EPSG:23700; see https://epsg.io/23700), which is the official national projected coordinate reference system (CRS) of Hungary, for details see Table 2.

**Table 2:** Technical properties of the common grid system and the produced grids.

Properties of the CRS of the common grid system	
CRS	HD72/EOV
EPSG	23700
Projection	Oblique Mercator
Datum	HD72
Spheroid	GRS1967
Units	meters
**Raster properties of the grids**	
Cell size	100 m
Columns	5120
Rows	3200
X_min_	426,000
X_max_	938,000
Y_min_	43,000
Y_max_	363,000

The spatial resolution of HU-EnviroGrids was selected to be 100 m, thus representing an optimal compromise between the coarse resolution climate variables and land surface variables derived from high-resolution satellite data, whilst remaining suitable for national-level analysis. All of the source data were reprojected to HD72/EOV at first, then resampled to 100 m spatial resolution (see Table 3), and finally the derived thematic variables were calculated based on the resampled data. The transformation, including reprojection, resampling, masking of environmental variables into the common grid system were performed using *terra* package^145^ in R software^146^ environment.

**Table 3:** Methods used for the resampling.

Environmental variables	Original resolution	Resampling method
topography	30 m	upscaling using area weighted average
hydrology:		
-surface hydrology	30 m	upscaling using area weighted average
-subsurface hydrology	100 m	not needed
climate	0.1° ~ 10 km	downscaling using cubic interpolation
biosphere & land surface	30 m	upscaling using area weighted average
pedosphere	100 m	not needed

The final dataset of HU-EnviroGrids is synthetized from several distinct data sources, incorporating both international data products and Hungarian national datasets. Due to differences in spatial extent, the Hungarian-specific data exhibited inconsistencies and gaps at the national borders. To resolve inconsistences and provide seamless dataset, edge-correction procedure was applied. All affected categorical data layers (Genetic soil type, Parent material, Topsoil texture) were processed using the *Shrink and expand* tool of SAGA GIS. An expansion operation was performed based on the majority value within a 5-pixel radius and subsequently, any redundant pixels generated beyond the national boundary were masked to ensure spatial consistency across all the variables. This approach ensured that the missing boundary cells were populated with the most frequent neighboring class, maintaining thematic integrity. The data gaps of GWTD, which is a continuous variable, were filled using *Close gaps* tool in SAGA GIS, utilizing spline interpolation with a tension threshold of 0.1 creating a natural transition.

## Data Records

Within the framework of HU-EnviroGrids, we provided a total of 147 environmental variables related to 5 domains organized into 49 files (Table 4). The dataset is freely available on Zenodo online repositiory (https://doi.org/10.5281/zenodo.17533785)^59^. The file format is GeoTIFF, with embedded georeferencing information (HD72/EOV, EPSG: 23700). For variables related to biosphere & land surface, it should be noted that not single- but multi-band data files were applied, containing 12 data layers for the satellite bands (Blue, Green, Red, NIR, SWIR1, and SWIR2) showing bare soil condition in arable lands and additional spectral indices (NDVI, TVI, SATVI, BI, NDSI, and LSWI) calculated based on the bare soil composite. For more details, see Table 4.

**Table 4:** Summary of the environmental variables incorporated in HU-EnviroGrids.

Related to	Environmental variable	Unit	Type
Topography	Altitude	m	continuous
	Aspect (classified)	—	categorical
	Aspect (non-classified)	radian	continuous
	Diurnal anisotropic heating	unitless	continuous
	General curvature	m^−1^	continuous
	Mid-Slope Position Index	unitless	continuous
	Multi-Resolution Ridge-Top Flatness	unitless	continuous
	Multi-Resolution Valley Bottom Flatness	unitless	continuous
	Multi-Scale Topographic Position Index	m	continuous
	Profile curvature	m^−1^	continuous
	Real surface area	m^2^	continuous
	Relief intensity	m	continuous
	Slope (degree)	degree	continuous
	Standardized height	m	continuous
	Surface roughness	m	continuous
	Terrain Ruggedness Index	m	continuous
	Topographic Position Index	m	continuous
	Total Curvature	m^−1^	continuous
	Valley depth	m	continuous
Hydrology	Channel network base level	m	continuous
	Ground water table depth	m	continuous
	Horizontal distance to channel network	m	continuous
	LS factor (Moore’s algorithm)	unitless	continuous
	Mass Balance Index	unitless	continuous
	SAGA Wetness Index	unitless	continuous
	Stream Power Index	unitless	continuous
	Topographic Wetness Index	unitless	continuous
	Vertical distance to channel network	m	continuous
Climate	Long-term mean annual (global) radiation (1991–2020)	W/m^2^	continuous
	Long-term mean seasonal (global) radiation (4 bands) (1991–2020)	W/m^2^	continuous
	Long-term mean annual evapotranspiration (1991–2020)	mm	continuous
	Long-term mean seasonal evapotranspiration (4 bands) (1991–2020)	mm	continuous
	Long-term mean annual precipitation (1991–2020)	mm	continuous
	Long-term mean seasonal precipitation (4 bands) (1991–2020)	mm	continuous
	Long-term mean annual temperature (1991–2020)	°C	continuous
	Long-term mean seasonal temperature (4 bands) (1991–2020)	°C	continuous
Biosphere & land surface	Long-term satellite spectral data (12 bands) (1991–2020)	unitless	continuous
	Short-term satellite spectral data (12 bands) (1991–1995)	unitless	continuous
	Short-term satellite spectral data (12 bands) (1996–2000)	unitless	continuous
	Short-term satellite spectral data (12 bands) (2001–2005)	unitless	continuous
	Short-term satellite spectral data (12 bands) (2006–2010)	unitless	continuous
	Short-term satellite spectral data (12 bands) (2011–2015)	unitless	continuous
	Short-term satellite spectral data (12 bands) (2016–2020)	unitless	continuous
	Short-term satellite spectral data (12 bands) (2021–2025)	unitless	continuous
Pedosphere	Genetic soil type	—	categorical
	Parent material	—	categorical
	Topsoil texture USDA (0–15 cm)	—	categorical

## Technical Validation

The HU-EnviroGrids dataset was derived from multiple, quality-controlled data sources, including FABDEM v1.2, the Meteorological Database of HungaroMet, E-OBS, USGS Landsat Archive (Collection-2, Level-2 data products) and DOSoReMI.hu. In order to ensure the reliability of the derived datasets of HU-EnviroGrids, a series of quality checks were performed on internal and thematic consistency, as well as spatial coverage. All of the variables completely cover the area of Hungary and have consistently the same number of valid pixels. Any missing values on the external boundaries and internal data gaps were filled. Out of range reflectance values were eliminated and the resultant gaps were also filled. The technical integrity of the dataset was verified by assessing the statistical distribution of all variables post-harmonization. The continuous variables remained within physically plausible ranges without mathematical artefacts (Table S1). The categorical variables were checked to ensure that the occurring class codes match the defined class schema: no undefined or missing class levels were found. Furthermore, the pixel counts of each level aligned with the expected thematic proportions in Hungary (Table S2).

## Usage Notes

It should be highlighted that HU-EnviroGrids provides a unique, comprehensive, and spatially exhaustive source of information on environmental conditions and factors across Hungary. To the best of our knowledge, no comparable dataset has previously been available for the entire territory of the country. Therefore, it can serve as a valuable resource for various disciplines that approach the environment systems in a holistic way. These include the disciplines of ecology, meteorology, forestry, hydrology, agriculture, and Earth and environmental sciences.

Recent advances in analytical and computational approaches (e.g., GeoAI and spatial foundation models) increasingly rely on the availability of large and well-structured environmental datasets. HU-EnviroGrids can therefore play an important role in enabling these data-intensive methods by providing consistent and spatially exhaustive information on Hungary’s environmental conditions.

The spatial resolution of HU-EnviroGrids (100 × 100 m) makes it suitable for a range of practical applications at national and regional scales, including rural development, land use planning, hydrological modelling, environmental hazards and risks assessment, climate modelling, sustainable agriculture, biogeochemical modelling, and ecosystem condition assessment. It can also be used to support evidence-based policy formulation and enhance public awareness of environmental issues. When considered in conjunction with other soil-related databases^20,22,126,147,148^, it provides a solid basis for, for instance, greenhouse gas (GHG) inventories aimed at mitigate climate change, soil mapping and process modelling to neutralize land degradation, and supporting the implementation of United Nations Convention to Combat Desertification (UNCCD). This dataset also serves as a gap-filling resource for international initiatives pursuing country-based or “bottom-up” spatial assessment and modelling such as EJP SOIL^149^.

However, it should be noted that the current spatial resolution may still be too coarse for applications at the local and settlement levels. Consequently, the utilization of the dataset is limited at finer spatial scales. Nevertheless, it can still be valuable for planning, calibrating and validating local-scale environmental monitoring systems. Potential users should also be aware that the accuracy of derived variables depends on the quality and resolution of the source data and the applied processing methods. While HU-EnviroGrids provides full national coverage, some local-scale variability may not be fully captured due to the 100 × 100 m resolution and input data heterogeneity.

HU-EnviroGrids has been designed with scalability in mind. In the future, we plan to extend it by developing finer spatial resolution layers and incorporating further thematic components. Such extensions will further enhance the dataset’s applicability and scientific value across disciplines.

## Supplementary information

Supplementary Table

## Data Availability

Data processing, including the derivation of topographic and hydrological variables, DEM conditioning and gap-filling was performed using standard tools in SAGA GIS v9.8.0^66^. The specific tools used are fully described in the Methods section. The calculation of climatic variables and spectral indices and data harmonization (reprojection, resampling and masking) was carried out in R v4.4.2^146^ using the standard functions of *terra* package^145^. The Google Earth Engine executable script can be accessed directly on the platform via the shared script repository at the following link: https://code.earthengine.google.com/?scriptPath = users%2Fmesserjanos%2Fshared%3ALandSat_composite_generation. The content of the script is also available to readers not registered on the platform in the Supplementary Material under the Code S1 subsection.
