# Supplementary material for: HU-EnviroGrids: A Gridded Environmental Dataset for Spatial Analysis and Modelling at country scale in Hungary
Source: Sci Data. 2026 Jun 9;13:1328. doi: 10.1038/s41597-026-07604-6 (PMC13590650; doi:10.1038/s41597-026-07604-6)
Supplement: Supplementary file 1 [file 41597_2026_7604_MOESM1_ESM.pdf]

Supplementary Table 1. Summary of raster value distributions for continuous type variables.

**Environmental variable**	**Total number of pixels**	**Valid pixels**	**Min.**	**Max.**	**Mean**	**Standard deviation**
Topography						
Altitude	16384000	9322943	32.41	1010.13	148.34	80.97
Aspect (non-classified)	16384000	9322943	0.00	6.28	3.05	1.75
Diurnal anisotropic heating	16384000	9322943	-0.60	0.51	0.00	0.04
General curvature	16384000	9322943	-0.01	0.01	0.00	0.00
Mid-Slope Position Index	16384000	9322943	0.00	0.97	0.36	0.22
Multi-Resolution Ridge-Top Flatness	16384000	9322943	0.00	9.62	2.31	2.23
Multi-Resolution Valley Bottom Flatness	16384000	9322943	0.00	9.91	4.56	3.60
Multi-Scale Topographic Position Index	16384000	9322943	-17.54	19.99	0.00	1.28
Profile curvature	16384000	9322943	-0.01	0.00	0.00	0.00
Real surface area	16384000	9322943	10000.00	13311.55	10019.14	69.28
Relief intensity	16384000	9322943	0.00	410.39	23.95	36.43
Slope (degree)	16384000	9322943	0.00	41.30	1.74	3.04
Standardized height	16384000	9322943	34.04	984.26	84.77	56.87
Surface roughness	16384000	9322943	0.00	14931.74	126.34	411.26
Terrain Ruggeddness Index	16384000	9322943	0.00	55.65	2.20	3.69
Topographic Position Index	16384000	9322943	0.00	0.00	0.00	0.00
Total Curvature	16384000	9322943	-108.37	117.51	0.00	6.79
Valley depth	16384000	9322943	2.91	435.03	33.34	32.64
Hydrology						
Channel network base level	16384000	9322943	71.05	905.48	135.81	62.41
Ground water table depth	16384000	9322943	0.00	19.20	3.35	1.44
Horizontal distance to channel network	16384000	9322943	0.00	10599.06	777.60	853.47
LS factor (Moore's algorithm)	16384000	9322943	0.09	94.89	1.09	2.33
Mass Balance Index	16384000	9322943	-0.79	1.27	0.01	0.07
SAGA Wetness Index	16384000	9322943	5.21	26.05	18.99	4.85

Stream Power Index		16384000	9322943	0.00	26581236.00	795.53	53197.20
Topographic Wetness Index		16384000	9322943	9.77	29.21	15.67	2.47
Vertical distance to channel network		16384000	9322943	-126.49	454.34	12.63	28.15
Climate							
Long-term mean annual (global) radiation (1991–2020)		16384000	9322943	134.24	152.44	144.78	3.95
Long-term mean seasonal (global) radiation (1991–2020)	DJF	16384000	9322943	44.13	56.88	51.30	2.92
	MAM	16384000	9322943	174.26	190.27	183.74	3.61
	JJA	16384000	9322943	224.13	251.16	240.31	5.93
	SON	16384000	9322943	90.81	111.14	101.57	4.51
Long-term mean annual evapotranspiration (1991–2020)		16384000	9322943	673.07	804.18	760.14	25.32
Long-term mean seasonal evapotranspiration (1991–2020)	DJF	16384000	9322943	35.50	52.14	44.49	3.60
	MAM	16384000	9322943	207.57	239.79	230.67	6.25
	JJA	16384000	9322943	319.82	374.51	356.36	10.70
	SON	16384000	9322943	110.10	141.98	128.61	6.59
Long-term mean annual precipitation (1991–2020)		16384000	9322943	528.53	866.68	613.28	60.73
Long-term mean seasonal precipitation (1991–2020)	DJF	16384000	9322943	82.76	159.32	115.04	12.41
	MAM	16384000	9322943	116.03	210.97	138.30	13.26
	JJA	16384000	9322943	172.56	315.39	201.96	19.91
	SON	16384000	9322943	127.74	233.04	157.97	24.52
Long-term mean annual temperature (1991–2020)		16384000	9322943	6.80	11.95	10.82	0.60
Long-term mean seasonal temperature (1991–2020)	DJF	16384000	9322943	-2.67	1.85	0.34	0.67
	MAM	16384000	9322943	6.30	12.25	11.13	0.60
	JJA	16384000	9322943	16.02	21.99	20.84	0.75
	SON	16384000	9322943	7.16	11.93	10.73	0.60
Biosphere & land surface							
Long-term satellite spectral data (1991–2020)	B	16384000	9322943	0.00	0.96	0.05	0.02
	G	16384000	9322943	0.01	0.97	0.08	0.02
	R	16384000	9322943	0.01	0.99	0.08	0.03

	NIR	16384000	9322943	0.00	0.98	0.23	0.08
	SWIR1	16384000	9322943	0.00	0.97	0.20	0.05
	SWIR2	16384000	9322943	0.00	0.94	0.14	0.05
	NDVI	16384000	9322943	-0.97	0.89	0.45	0.25
	TVI	16384000	9322943	0.00	117.80	96.47	14.01
	SATVI	16384000	9322943	-0.95	0.52	0.16	0.07
	BI	16384000	9322943	0.02	0.67	0.06	0.02
	NDSI	16384000	9322943	-88.76	97.24	-45.02	24.64
	LSWI	16384000	9322943	-0.95	0.98	0.06	0.21
Short-term satellite spectral data (1991–1995)	B	16384000	9322943	0.01	1.00	0.06	0.02
	G	16384000	9322943	0.02	0.98	0.09	0.03
	R	16384000	9322943	0.02	1.00	0.09	0.03
	NIR	16384000	9322943	0.03	1.00	0.24	0.08
	SWIR1	16384000	9322943	0.01	0.99	0.20	0.05
	SWIR2	16384000	9322943	0.00	0.99	0.14	0.05
	NDVI	16384000	9322943	-0.70	0.87	0.41	0.22
	TVI	16384000	9322943	0.00	116.95	94.71	11.94
	SATVI	16384000	9322943	-1.27	0.74	0.14	0.06
	BI	16384000	9322943	0.02	0.69	0.06	0.02
	NDSI	16384000	9322943	-86.78	70.32	-41.13	21.97
	LSWI	16384000	9322943	-0.64	0.87	0.06	0.20
Short-term satellite spectral data (1996–2000)	B	16384000	9322943	0.00	0.93	0.06	0.02
	G	16384000	9322943	0.01	1.00	0.08	0.02
	R	16384000	9322943	0.01	1.00	0.08	0.03
	NIR	16384000	9322943	0.02	1.00	0.23	0.08
	SWIR1	16384000	9322943	0.00	0.99	0.20	0.05
	SWIR2	16384000	9322943	0.00	0.96	0.14	0.06
	NDVI	16384000	9322943	-0.76	0.87	0.43	0.23
	TVI	16384000	9322943	0.00	117.10	95.32	13.37
	SATVI	16384000	9322943	-1.08	0.41	0.15	0.06
	BI	16384000	9322943	0.01	0.70	0.06	0.02
	NDSI	16384000	9322943	-87.13	75.58	-42.64	23.25

	LSWI	16384000	9322943	-0.74	0.89	0.05	0.21
Short-term satellite spectral data (2001–2005)	B	16384000	9322943	0.00	1.00	0.06	0.03
	G	16384000	9322943	0.00	1.00	0.08	0.03
	R	16384000	9322943	0.00	1.00	0.08	0.04
	NIR	16384000	9322943	0.00	1.00	0.23	0.08
	SWIR1	16384000	9322943	0.00	1.00	0.20	0.05
	SWIR2	16384000	9322943	0.00	1.00	0.14	0.06
	NDVI	16384000	9322943	-1.00	0.88	0.44	0.24
	TVI	16384000	9322943	0.00	117.47	96.04	13.70
	SATVI	16384000	9322943	-1.16	0.70	0.16	0.07
	BI	16384000	9322943	0.00	0.70	0.06	0.03
	NDSI	16384000	9322943	-87.99	99.88	-44.10	23.89
	LSWI	16384000	9322943	-1.00	1.00	0.05	0.21
Short-term satellite spectral data (2006–2010)	B	16384000	9322943	0.00	1.00	0.05	0.02
	G	16384000	9322943	0.00	0.99	0.08	0.03
	R	16384000	9322943	0.01	1.00	0.08	0.04
	NIR	16384000	9322943	0.00	1.00	0.24	0.08
	SWIR1	16384000	9322943	0.00	1.00	0.21	0.05
	SWIR2	16384000	9322943	0.00	0.99	0.14	0.06
	NDVI	16384000	9322943	-1.00	0.88	0.46	0.25
	TVI	16384000	9322943	0.00	117.49	96.91	13.69
	SATVI	16384000	9322943	-1.11	0.58	0.17	0.07
	BI	16384000	9322943	0.01	0.70	0.06	0.02
	NDSI	16384000	9322943	-88.04	99.76	-45.79	24.65
	LSWI	16384000	9322943	-1.00	0.89	0.06	0.21
Short-term satellite spectral data (2011–2015)	B	16384000	9322943	0.00	0.98	0.05	0.02
	G	16384000	9322943	0.00	0.93	0.07	0.02
	R	16384000	9322943	0.01	0.99	0.08	0.04
	NIR	16384000	9322943	0.00	0.99	0.24	0.08
	SWIR1	16384000	9322943	0.00	0.95	0.21	0.05
	SWIR2	16384000	9322943	0.00	0.99	0.14	0.06
	NDVI	16384000	9322943	-0.83	0.90	0.47	0.26

	TVI	16384000	9322943	0.00	118.39	97.45	14.95
	SATVI	16384000	9322943	-0.89	0.62	0.18	0.07
	BI	16384000	9322943	0.01	0.66	0.05	0.02
	NDSI	16384000	9322943	-90.15	83.37	-47.17	26.22
	LSWI	16384000	9322943	-0.64	0.90	0.06	0.22
Short-term satellite spectral data (2016–2020)	B	16384000	9322943	0.00	0.96	0.05	0.02
	G	16384000	9322943	0.00	1.00	0.07	0.03
	R	16384000	9322943	0.00	1.00	0.08	0.04
	NIR	16384000	9322943	0.00	1.00	0.24	0.08
	SWIR1	16384000	9322943	0.00	0.99	0.20	0.05
	SWIR2	16384000	9322943	0.00	0.99	0.13	0.06
	NDVI	16384000	9322943	-1.00	0.90	0.48	0.26
	TVI	16384000	9322943	0.00	118.51	98.13	14.93
	SATVI	16384000	9322943	-1.16	0.46	0.17	0.07
	BI	16384000	9322943	0.00	0.68	0.05	0.02
	NDSI	16384000	9322943	-90.44	100.00	-48.41	26.25
	LSWI	16384000	9322943	-1.00	0.99	0.07	0.21
Short-term satellite spectral data (2021–2025)	B	16384000	9322943	0.00	1.00	0.05	0.03
	G	16384000	9322943	0.00	0.99	0.07	0.03
	R	16384000	9322943	0.00	1.00	0.08	0.05
	NIR	16384000	9322943	0.00	1.00	0.25	0.07
	SWIR1	16384000	9322943	0.00	1.00	0.21	0.06
	SWIR2	16384000	9322943	0.00	1.00	0.14	0.07
	NDVI	16384000	9322943	-1.00	0.93	0.49	0.26
	TVI	16384000	9322943	0.00	119.39	98.31	14.63
	SATVI	16384000	9322943	-1.22	0.58	0.18	0.07
	BI	16384000	9322943	0.00	0.70	0.06	0.03
	NDSI	16384000	9322943	-92.53	100.00	-48.66	26.30
	LSWI	16384000	9322943	-1.00	0.98	0.07	0.21

Supplementary Table 2. Class composition and frequency statistics of categorical type variables.

Environmental variable	Class level	Category name	Pixel count	Percentage [%]
Genetic soil type	10	Stony skeletal soils	6253	0.07
	20	Gravelly skeletal soils	76239	0.82
	30	Barren earths	163156	1.75
	40	Shifting sand	269630	2.89
	50	Humic sandy soils	601043	6.45
	60	Humus-carbonate soils	50309	0.54
	70	Rendzinas	206670	2.22
	80	Erubase soils	21533	0.23
	90	Acidic, non-podzolic brown forest soils	38104	0.41
	91	Rankers	153744	1.65
	100	Podzolic brown forest soils	111476	1.20
	110	Brown forest soils with clay accumulation	595868	6.39
	120	Stagnant brown forest soils	155107	1.66
	130	Brown earths	1090742	11.70
	132	Brown earths on sand	13675	0.15
	140	Banded brown forest soils	133553	1.43
	150	Brown forest soils with carbonate residues	65161	0.70
	160	Chernozem brown forest soils	228733	2.45
	180	Leached chernozems	38055	0.41
	190	Pseudomyceliar chernozems	636845	6.83
	200	Meadow chernozems	760083	8.15
	210	Alluvial chernozems	49920	0.54
	220	Solonchaks	25933	0.28
	230	Solonchak-solonetzes	14441	0.15
	240	Meadow solonetzes	219671	2.36
	250	Steppe meadow solonetzes	117646	1.26
	270	Human-induced salt-affected soils	8211	0.09
	280	Solonchak meadow soils	4658	0.05

	290	Solonetzic meadow soils	234007	2.51
	300	Typic meadow soils	1507516	16.17
	303	Meadow soils with deep salt accumulation	228639	2.45
	310	Alluvial meadow soils	537771	5.77
	320	Peaty meadow soils	80523	0.86
	330	Chernozem meadow soils	86253	0.93
	340	Sphagnum peats	612	0.01
	350	Meadow peat soils	42089	0.45
	360	Ameliorated peats	6292	0.07
	370	Soils of swampy forests	111200	1.19
	380	Raw alluvial soils	66257	0.71
	390	Humic alluvial soils	417082	4.47
	400	Colluvial soils	148243	1.59
		Valid pixels	*9322943*	100
		Total number of pixels	*16384000*	
Parent material	1	Intermediate and basic igneous rocks	94668	1.02
	2	Tuffs and volcanic agglomerate	100918	1.08
	3	Sandstone, conglomerate	108393	1.16
	4	Claystone, aleurolite	173683	1.86
	5	Calcareous and carbonate rocks	203169	2.18
	6	Rock fragments, gravel, sand (coarse sediments; colluvial, fluvial, proluvial, lacustrine)	1051591	11.28
	7	Clay, aleurit, sand (mixed sediments; colluvial, fluvial)	1193694	12.80
	8	Clay, aleurit (fine sediments; fluvial, colluvial, lacustrine, paludal)	2383417	25.57
	9	Loess (eolian, hydroeolian)	2317069	24.85
	10	Loess sand (eolian)	172361	1.85
	11	Shifting sand	1192095	12.79
	12	Peat bog	57762	0.62
	13	Miscellaneous	98341	1.05
	14	Water	175782	1.89
		Valid pixels	*9322943*	100

		Total number of pixels	*16384000*	
Topsoil texture USDA (0–15 cm)	1	Clay	272387	2.92
	2	Silty clay	620038	6.65
	3	Silty clay loam	1177764	12.63
	4	Sandy clay	136	0.00
	5	Sandy clay loam	88105	0.95
	6	Clay loam	833869	8.94
	7	Silt	1784	0.02
	8	Silt loam	1931200	20.71
	9	Loam	2257454	24.21
	10	Sand	711540	7.63
	11	Loamy sand	364238	3.91
	12	Sandy loam	1064428	11.42
		Valid pixels	*9322943*	100
		Total number of pixels	*16384000*	
Aspect (classified)	0	flat	63719	0.68
	1	North	890947	9.56
	2	Northeast	1198567	12.86
	3	East	1394127	14.95
	4	Southeast	1116723	11.98
	5	South	1094823	11.74
	6	Southwest	1305910	14.01
	7	West	1305631	14.00
	8	Northwest	952496	10.22
		Valid pixels	*9322943*	100
		Total number of pixels	*16384000*	

Supplementary code 1. Google Earth Engine script to generate the biosphere and land surface related data layers based on the images of LandSat satellite series.

```
////////////////////////////////////////////////////////////////////////////////
// Imported data layers from personal assets
// Raster layer of 100-meter resolution grid to use as mask layer for the area of Hungary
var grid = ee.Image("projects/ee-messerjanos-430909/assets/HUgridsEOV/HU_EOV_100m"),
// Raster layer of land-use categories based on the Ecosystem Map of Hungary
landuse = ee.Image("projects/ee-messerjanos-430909/assets/landuse/LU_HUN");

////////////////////////////////////////////////////////////////////////////////
// Google Earth Engine Javascript-based script to generate
// composite images with spectral bands and several different
// indices based on images acquired by LandSat satellite family.

// Investigated time period can be defined freely by adding start and end years.

// Developer: János Mészáros, 2025
// Email: meszaros.janos@atk.hun-ren.hu
////////////////////////////////////////////////////////////////////////////////

// Date variables to define period
var yearStart = 1991;
var yearEnd = 1995;

////////////////////////////////////////////////////////////////////////////////
// LandSat image collections import
var ls91 = ee.ImageCollection("LANDSAT/LC09/C02/T1_L2");
var ls92 = ee.ImageCollection("LANDSAT/LC09/C02/T2_L2");
var ls81 = ee.ImageCollection("LANDSAT/LC08/C02/T1_L2");
var ls82 = ee.ImageCollection("LANDSAT/LC08/C02/T2_L2");
var ls71 = ee.ImageCollection("LANDSAT/LE07/C02/T1_L2");
var ls72 = ee.ImageCollection("LANDSAT/LE07/C02/T2_L2");
var ls51 = ee.ImageCollection("LANDSAT/LT05/C02/T1_L2");
var ls52 = ee.ImageCollection("LANDSAT/LT05/C02/T2_L2");
var ls41 = ee.ImageCollection("LANDSAT/LT04/C02/T1_L2");
var ls42 = ee.ImageCollection("LANDSAT/LT04/C02/T2_L2");


////////////////////////////////////////////////////////////////////////////////
// Functions for LandSat image processing
// Function for cloud masking LandSat-8 and -9 images
function cm89(image) {
 // Bits 4 and 3 are cloud shadow and cloud, respectively
 var cloudShadowBitMask = (1 << 4);
 var cloudsBitMask = (1 << 3);
 // Get the QA band
 var qa = image.select('QA_PIXEL');
 // Both flags are set to zero, indicating clear conditions
 var cmask = qa.bitwiseAnd(cloudShadowBitMask).eq(0)
 .and(qa.bitwiseAnd(cloudsBitMask).eq(0));
```

```
  image = image.mask(cmask).clip(grid.geometry()).updateMask(grid);

  image = image.select('SR_B.').multiply(0.0000275).add(-0.2);

  return image.select(['SR_B2', 'SR_B3', 'SR_B4', 'SR_B5', 'SR_B6', 'SR_B7'],
    ['B', 'G', 'R', 'NIR', 'SWIR1', 'SWIR2']);
}

// Function for cloud masking  LandSat-5 images
function cm457(image) {
  // Bits 4 and 3 are cloud shadow and cloud, respectively
  var cloudShadowBitMask = (1 << 4);
  var cloudsBitMask = (1 << 3);
  // Get the pixel QA band
  var qa = image.select('QA_PIXEL');
  // Both flags should be set to zero, indicating clear conditions
  var cmask = qa.bitwiseAnd(cloudShadowBitMask).eq(0)
  .and(qa.bitwiseAnd(cloudsBitMask).eq(0));
  image = image.mask(cmask).clip(grid.geometry()).updateMask(grid);

  image = image.select('SR_B.').multiply(0.0000275).add(-0.2);

  return image.select(['SR_B1', 'SR_B2', 'SR_B3', 'SR_B4', 'SR_B5', 'SR_B7'],
    ['B', 'G', 'R', 'NIR', 'SWIR1', 'SWIR2']);
}

// Function to mask arable land pixels and filtering them for bare soil condition
function arableland_mask(image) {
  var lumask = landuse.select('b1').eq(2100);
  var ndvi = image.normalizedDifference(['NIR', 'R']);
  var nbr = image.normalizedDifference(['NIR', 'SWIR2']);
  var spmask = ndvi.lt(0.25).and(nbr.lt(0.2));
  return image.updateMask(lumask).updateMask(spmask);
}

// Function to mask non-arable land pixels
function nonarableland_mask(image) {
  var lumask = landuse.select('b1').eq(2100).not();
  return image.updateMask(lumask);
}


/////////////////////////////////////////////////////////////////////////////////
// Satellite image filtering
// Adding months and days to the defined period
var dateStart = ee.Date.fromYMD(yearStart, 1, 1);
var dateEnd = ee.Date.fromYMD(yearEnd, 12, 31);

// Filtering each LandSat satellite collection
// LandSat-9 images
var ls91f = ls91.filterDate(dateStart, dateEnd)
  .filterBounds(grid.geometry())
```

```
  .filterMetadata('CLOUD_COVER_LAND',"less_than", 50)
  .map(cm89);

// LandSat-9 images
var ls92f = ls92.filterDate(dateStart, dateEnd)
  .filterBounds(grid.geometry())
  .filterMetadata('CLOUD_COVER_LAND',"less_than", 50)
  .map(cm89);

// LandSat-8 images
var ls81f = ls81.filterDate(dateStart, dateEnd)
  .filterBounds(grid.geometry())
  .filterMetadata('CLOUD_COVER_LAND',"less_than", 50)
  .map(cm89);

// LandSat-8 images
var ls82f = ls82.filterDate(dateStart, dateEnd)
  .filterBounds(grid.geometry())
  .filterMetadata('CLOUD_COVER_LAND',"less_than", 50)
  .map(cm89);

// LandSat-7 images
var ls71f = ls71.filterDate(dateStart, dateEnd)
  .filterBounds(grid.geometry())
  .filterMetadata('CLOUD_COVER_LAND',"less_than", 50)
  .map(cm457);

// LandSat-7 images
var ls72f = ls72.filterDate(dateStart, dateEnd)
  .filterBounds(grid.geometry())
  .filterMetadata('CLOUD_COVER_LAND',"less_than", 50)
  .map(cm457);

// LandSat-5 images
var ls51f = ls51.filterDate(dateStart, dateEnd)
  .filterBounds(grid.geometry())
  .filterMetadata('CLOUD_COVER_LAND',"less_than", 50)
  .map(cm457);

// LandSat-5 images
var ls52f = ls52.filterDate(dateStart, dateEnd)
  .filterBounds(grid.geometry())
  .filterMetadata('CLOUD_COVER_LAND',"less_than", 50)
  .map(cm457);

// LandSat-4 images
var ls41f = ls41.filterDate(dateStart, dateEnd)
  .filterBounds(grid.geometry())
  .filterMetadata('CLOUD_COVER_LAND',"less_than", 50)
  .map(cm457);

// LandSat-4 images
```

```
var ls42f = ls42.filterDate(dateStart, dateEnd)
  .filterBounds(grid.geometry())
  .filterMetadata('CLOUD_COVER_LAND',"less_than", 50)
  .map(cm457);

// Merging filtered images into one collection
var lsmerge = ls91f.merge(ls92f).merge(ls81f).merge(ls82f)
      .merge(ls71f).merge(ls72f).merge(ls51f).merge(ls52f)
      .merge(ls41f).merge(ls42f);

// Printing total number of used satellite images into Console
print(ee.String('Number of used images: ').cat(lsmerge.size()));


////////////////////////////////////////////////////////////////////////////////
// Creating bare soil composite image for both land-use group
// Bare soil median image for arable lands
var lsbs = lsmerge.map(arableland_mask).median();
// Non-arable land areas median image
var lsabs = lsmerge.map(nonarableland_mask).median();
// Merging two images as image collection
var lsbsc = ee.ImageCollection([lsbs, lsabs]);

// Mosaic image created from previous image collection
var lsm = lsbsc.mosaic();

// Define the projection and scale of the grid mask layer
var areaProjection = grid.projection();
var areaScale = 100; // meters

// Resample the mosaic to fit the pixel grid of the area mask layer
var lsm = lsm.reproject({
  crs: areaProjection,
  scale: areaScale
});


////////////////////////////////////////////////////////////////////////////////
// Calculating spectral indices as additional bands
// NDVI
var ndvi = lsm.expression(
  'NDVI = (B01 - B02) / (B01 + B02)', {
    'B01': lsm.select('NIR'),
    'B02': lsm.select('R')
});
lsm = lsm.addBands(ndvi);
// TVI
var tvi = lsm.expression(
  'TVI = ((B01 - B02) / (B01 + B02) + 0.5)**0.5 * 100', {
    'B01': lsm.select('NIR'),
    'B02': lsm.select('R')
});
```

```
lsm = lsm.addBands(tvi);
// SATVI
var satvi = lsm.expression(
  'SATVI = ((B01 - B02) / (B01 + B02 + 1)) * 2 - (B03 / 2)', {
    'B01': lsm.select('SWIR1'),
    'B02': lsm.select('R'),
    'B03': lsm.select('SWIR2')
});
lsm = lsm.addBands(satvi);
// BI - brightness index Escadafal, 1989
var bi = lsm.expression(
  'BI = ((B01 * B01) + (B02 * B02))**0.5 / 2', {
    'B01': lsm.select('R'),
    'B02': lsm.select('G')
});
lsm = lsm.addBands(bi);
// NDSI - normalized difference salinity index Tripathi et al., 1997
var ndsi = lsm.expression(
    'NDSI = ((B01 - B02) / (B01 + B02))*100', {
      'B01': lsm.select('R'),
      'B02': lsm.select('NIR')
});
lsm = lsm.addBands(ndsi);
// LSWI - land surface water index
var lswi = lsm.expression(
  'LSWI = (B01 - B02) / (B01 + B02)', {
    'B01': lsm.select('NIR'),
    'B02': lsm.select('SWIR1')
});
lsm = lsm.addBands(lswi);


////////////////////////////////////////////////////////////////////////////////
// Visualizing true-color satellite image
// Color palette definition
var LSvis = {
  min: 0.0,
  max: 0.2,
  bands: ['R', 'G', 'B'],
};

// Map viewer options
Map.setOptions("ROADMAP");
Map.centerObject(grid, 10);

// Adding new image layer to the main map view
Map.addLayer(lsm, LSvis, 'Landsat composite image - true-color');
```
